# Hybrid Analysis of Videoconference Technology Use by Aging-in-Place Organizations to Promote Social Engagement for Older Adults: A Scoping Review with Latent Topic Modeling

**DOI:** 10.3390/healthcare13233031

**Published:** 2025-11-24

**Authors:** John Alagood, William D. Senn, Gayle Prybutok

**Affiliations:** 1 Department of Management and Marketing, Texas Woman’s University, Denton, TX 76204, USA; 2 Department of Marketing and Computer Information Systems, Tarleton State University, Stephenville, TX 76402, USA; wsenn@tarleton.edu; 3 Department of Rehabilitation and Health Services, University of North Texas, Denton, TX 76201, USA; gayle.prybutok@unt.edu

**Keywords:** older adults, aging in place, social engagement, loneliness, social isolation, videoconferencing, digital accessibility, ICT adoption, HCI, social technology, PRISMA

## Abstract

**Background/Objectives**: Loneliness and social isolation are common among older adults and linked to adverse health outcomes. Videoconferencing can support social connections, but the role of aging-in-place organizations (AIPOs), such as senior centers and Area Agencies on Aging, in facilitating adoption is poorly understood. This review examined how AIPOs use relational videoconferencing to promote social engagement among older adults. **Methods**: We applied a hybrid methodology combining a scoping review with latent topic modeling to contextualize and analyze the evidence base. Exploratory searches revealed limited literature specifically addressing AIPO involvement; therefore, we first conducted latent topic modeling of the broader literature on social videoconferencing among older adults to establish a thematic foundation for the subsequent PRISMA-guided scoping review. Thematic analysis of this broader corpus, identified through 2021 database searches, applied Latent Dirichlet Allocation (LDA) to a collection of peer-reviewed articles. Subsequent refinement of this corpus by removing non-primary research and non-AIPO records produced the narrower PRISMA subset used for the scoping review. The scoping review followed JBI guidelines and was based on database searches (EBSCOhost: MEDLINE, AgeLine, SocINDEX, Health Source: Nursing/Academic Edition, and Family & Society Studies Worldwide; ProQuest Social Science Premium Collection; and PubMed, including MEDLINE, PMC, and in-process content) for peer-reviewed studies published between 2011 and 2025. Inclusion criteria required primary research involving adults aged 65 years or older, use of videoconferencing technology for social engagement, and reference to AIPOs or analogous community-based aging services. The protocol was post-registered with the Open Science Framework. **Results**: The LDA analysis of 101 peer-reviewed articles identified six latent themes describing the broader research landscape: problem of isolation, character of socialization, physical health, technology as intervention, technology as social medium, and supportive environments. This thematic framework informed the scoping review, which screened 1908 records and retained 25 publications (representing 24 unique studies) explicitly referencing AIPO involvement in relational videoconferencing. Only one study predated COVID-19. Mapping these studies to the LDA-derived themes revealed the least consistent coverage to be in supportive environments and physical health, particularly among AIPOs other than senior or community centers. **Conclusions**: Relational videoconferencing has potential to sustain and expand older adults’ social connections, but evidence mapped through the scoping review shows that documentation of how AIPOs support adoption is sparse. The hybrid approach advances understanding of videoconferencing in aging contexts and identifies priorities for documenting, comparing, and refining AIPO practices to inform future interventions and policy.

## 1. Introduction

### 1.1. Purpose

The purpose of this study is to examine how aging-in-place organizations use relational videoconferencing to foster social engagement among older adults. It contributes to the understanding of how such organizations can implement technology to foster social connection and potentially reduce loneliness among older adults in their service communities. The study draws on a scoping review and topic modeling analysis to map the existing evidence, identify gaps, and explore how AIPOs implement videoconferencing to support older adults’ social engagement.

### 1.2. Background

#### 1.2.1. Social Isolation and Loneliness

Loneliness and social isolation are extremely prevalent among older adults and correlate with mortality and diverse health risks, including conditions such as schizophrenia, tuberculosis, and accidents [1,2,3]. Seminal work performed by House et al. [3] compared the health consequences of inadequate social relationships with those of cigarette smoking, obesity, and heart disease. Although the terms “loneliness” and “social isolation” are sometimes used without distinction, they represent different concepts. Loneliness is more subjective, based on the felt experience of the individual, whereas social isolation is more tangible, related to the size of and activity within a social network [4,5]. Meta-analysis of studies addressing both social isolation and loneliness (also known as objective and perceived social isolation, respectively) has found limited correlation between them and suggested they may influence health through separate pathways [5].

#### 1.2.2. Videoconferencing Technology for Social Connection

Videoconferencing, as a form of social technology, represents a critical interface between humans and machines that enables older adults to sustain and expand meaningful relationships. Within the broader category of information and communication technology (ICT), social technology emphasizes ease of use, accessibility, and intuitive design to foster connectedness [6]. Videoconferencing integrates audio, visual, and interactive elements that allow communication to approximate in-person social cues, supporting engagement in family interactions, peer groups, and community activities such as educational and exercise programs. Studies have consistently found that social use of digital technologies, including videoconferencing, can mitigate both loneliness and social isolation [7,8], with progressively older generations showing increasing adoption of such tools.

Under Media Richness Theory [9], communication media differ in their capacity to convey shared understanding, with “richer” media transmitting more nonverbal, auditory, and contextual cues. Videoconferencing is among the richest available digital media, offering advantages over phone or text communication through its ability to convey facial expressions, tone, and gestures that foster familiarity and emotional closeness [10,11]. The presence of visual and auditory feedback enhances perceived immediacy, emotional resonance, and psychological proximity, reducing social distance and supporting sustained engagement [12].

However, the relational benefits of videoconferencing depend heavily on factors influencing adoption and sustained use. Older adults’ information and communication technology (ICT) skills, cognitive load, and comfort with being observed all shape satisfaction and long-term engagement. Studies comparing videoconferencing with telephone or text-based interaction have found mixed outcomes, suggesting that cognitive demand and self-consciousness during use may moderate its benefits [11,13]. Tools that assess older adults’ digital abilities, such as those developed by Yu and Chao [14], highlight how tailored assessment and training can help address skill gaps and increase adoption. Moreover, sustained use of digital tools among older adults has been shown to depend on structured instruction and facilitation—underscoring the role of aging-in-place organizations (AIPOs) and other community supports in building digital confidence [15].

When usability, support, and motivation align, videoconferencing can meaningfully reduce loneliness and strengthen social ties despite its technical and emotional demands. Even when challenges arise, the ability to see and interact with others in real time provides unique emotional and psychological benefits, reinforcing its promise as a tool for promoting social engagement and well-being among older adults [16].

#### 1.2.3. Older Adults

For the purposes of this study, older adults are defined as persons aged 65 and older, a commonly used distinction in literature reviews and consistent with the description used by the National Institute on Aging [17,18]. Surveys consistently show that most older adults wish to age “in place,” remaining in familiar and comfortable environments [19]. Yet this population is generally less likely to adopt information and communication technologies (ICTs) [20,21,22,23] and is vulnerable to the compounding effects of loneliness and depression [24].

#### 1.2.4. Aging-in-Place Organizations

Aging-in-place organizations (AIPOs) are community-based entities that provide programs and services enabling older adults to remain safely and independently in their homes and communities. Services include care coordination, health navigation, social programming, and technology support. In the United States, many AIPOs operate under the framework established by the Older Americans Act [25], which created Area Agencies on Aging, senior centers, and related programs. Internationally, similar organizations exist under different models, such as voluntary sector organizations in the United Kingdom, community support services in Australia, and municipal aging services in Nordic countries.

In this article, we use the umbrella term AIPOs to refer to organizations that coordinate or deliver long-term services and supports (LTSS), information/navigation, and social-connection programs. We anchor this construct in the Older Americans Act (OAA) Aging Network—including State Units on Aging, Area Agencies on Aging, OAA service providers, Title VI tribal aging programs, multipurpose senior centers, and Aging & Disability Resource Centers (ADRCs). The federal mandate for U.S. senior centers, for example, explicitly includes socialization of older adults [25]. We also use the term AIPO broadly to include analogous community-based organizations, regardless of country, that share an aging-in-place mission. Congregate living environments, however, were excluded to maintain focus on the relational needs of community-dwelling older adults.

Together, older adults’ desire to remain in place and the supportive role of AIPOs set the stage for understanding recent changes in technology use. During the COVID-19 pandemic, this alignment of needs and services became especially relevant, as quarantines compounded risks of isolation while simultaneously accelerating adoption of relational technologies. Senior centers and other AIPOs sought to engage their members through relational videoconference tools [26,27], facing adoption challenges moderated by age [28] and potentially mediated by lifestyle [29].

Table 1 summarizes the key terms and operational definitions used throughout this study, providing conceptual clarity for constructs such as loneliness, social isolation, social technology, and aging-in-place organizations (AIPOs).

### 1.3. Study Contribution and Impact

This study not only synthesizes existing literature on AIPO-led relational videoconferencing but also illustrates the value of a hybrid methodology combining scoping review with latent topic modeling. This approach integrates explicit, exploratory, question-driven synthesis with data-driven thematic mapping, revealing both overt findings and latent patterns. The resulting insights inform practice, policy, and research while offering a replicable model for future hybrid studies in aging, social technology, and health domains.

## 2. Materials and Methods

A scoping review framework was selected for this study in response to the limited, heterogeneous, and emergent nature of the literature on videoconferencing by aging-in-place organizations (AIPOs). This approach allows for systematic mapping of evidence, identification of research gaps, and characterization of implementation across diverse contexts, yielding insights into a corpus not yet sufficiently robust for a traditional systematic review. The review followed the framework of Arksey and O’Malley (2005) [35], with refinements from Peters et al. (2020) [36], and is reported according to PRISMA-ScR guidelines (Tricco et al., 2018) [37].

An initial exploratory search revealed very few studies specifically focused on AIPO adoption of relational videoconferencing. To establish a contextual foundation for the scoping review, we therefore conducted latent topic modeling before the formal review process, applying an unsupervised Latent Dirichlet Allocation (LDA) [38] analysis to the broader corpus of literature identified through 2021 database searches. This corpus was identified during the overall search process and subsequently refined to produce the scoping review dataset by removing non-primary research and non-AIPO records. The LDA stage provided a thematic context that informed the structure and focus of the subsequent PRISMA-guided scoping review, identifying patterns and thematic relationships that may influence older adults’ social engagement and associated health outcomes.

The review addressed two complementary aims, integrating latent topic modeling with scoping review procedures. LDA provided a thematic overview of the broader corpus, while the scoping review synthesized the subset of primary research focusing on AIPOs and analogous community-based organizations. This approach allowed us to integrate a transparent, exploratory synthesis with a data-driven map of the field, as recommended for complex health and human–computer interaction (HCI) domains [39,40].

Our rationale parallels the approach of Greenhalgh et al. (2017) [41] in developing their health technology adoption framework (NASSS), where a scoping review was integrated with a second approach (data-driven, inductive case studies) that allowed them to address the topic’s inherent complexity. Similarly, our integration of scoping review and latent thematic modeling reflects the need for multi-pronged analytic strategies in complex domains, providing insights relevant to interventions that support older adults’ well-being. Unsupervised models reveal structure but do not appraise relevance to a targeted context; scoping review procedures do. Combining both methods allows us to map the field while maintaining context-specific relevance to health and social engagement outcomes.

### 2.1. Scoping Review

An initial literature search was conducted in November 2021 and updated in October 2025 using targeted databases to identify articles addressing the role of aging-in-place organizations in adopting relational videoconference technology by older adults.

#### 2.1.1. Eligibility Criteria

Consistent with JBI guidance [36], inclusion criteria reflected the population (older adults aged 65 years or above), concept (relational videoconferencing for social engagement), and context (AIPOs and analogous community-based aging services). Results were limited to English-language, peer-reviewed, primary-research, journal articles published between 2011 and 2025, inclusive, that addressed the role of aging-in-place organizations in social engagement of older adults aged 65 years and above using videoconferencing technology. Studies were limited to those published in English due to resource constraints and the linguistic capacity of the research team. Additionally, grey literature was not included in this review. This decision reflects our focus on peer-reviewed research that has undergone editorial evaluation and the need to maintain feasibility within available resources.

This period encompasses both the early mainstream adoption of platforms like Apple FaceTime and Zoom, as well as the significant inflection point driven by the COVID-19 pandemic beginning in early 2020. For peer-reviewed articles that addressed social engagement of older adults using videoconferencing technology, the full text was evaluated, and those that explicitly considered the role of aging-in-place organizations were included in the final set.

#### 2.1.2. Information Sources

Seven databases were queried with database selection informed by expert opinion regarding relevance to the subject matter. The databases included were EBSCOhost’s MEDLINE, AgeLine, SocINDEX, Health Source: Nursing/Academic Edition, and Family & Society Studies Worldwide; the ProQuest Social Science Premium Collection; and PubMed’s MEDLINE, including PMC and in-process content.

#### 2.1.3. Search Strategies

Database queries were determined based on a cursory literature review and then refined through discussion with experts in healthcare, aging, and technology. Boolean logic and truncation were used to ensure a comprehensive yet targeted search.

For each database, a search strategy was developed that applied the following search categories within the context of the database search tools:

- Older adults;
- Social technology;
- Social connections;
- Social isolation and loneliness.

**Figure 1:**
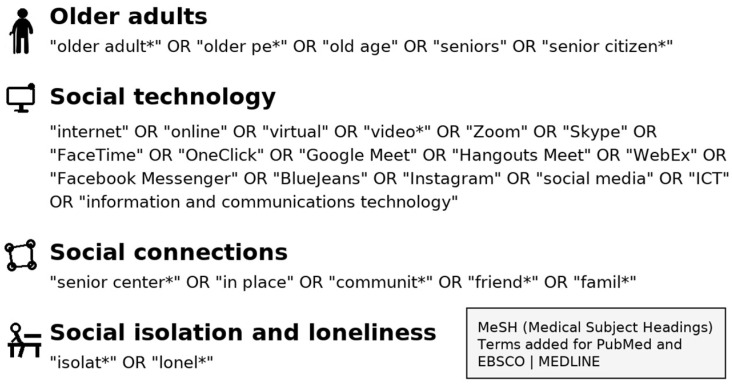
Scoping review search terms. The asterisk (*) indicates a truncation wildcard used to include various endings of the word.

These four search topics were combined so that each of the four categories was required to be included (AND logic), but any term within each of the four (as described in Figure 1) would satisfy the presence of that category (OR logic).

The scoping review search strategy table (Supplementary Table S1) documents for each database several categories described by the authors, including: search ID, database name, search dates and date ranges covered, exact search syntax, fields searched, search options selected, and number of records identified. The Social Science Premium Collection used for the 2021 search has been discontinued in that format and was therefore not used for the 2025 update. This database resulted in just 2.0% of all records identified in the 2021 search, and no incremental reports were included.

#### 2.1.4. Search Queries

A search query was designed and performed separately for each database, including for the EBSCOhost platform databases, where the option to search simultaneously was not employed.

The following example was executed in ProQuest:

(“older adult*” OR “older pe*” OR “old age” OR “seniors” OR “senior citizen*”) AND (“internet” OR online OR “virtual” OR video* OR Zoom OR Skype OR FaceTime OR OneClick OR “Google Meet” OR “Hangouts Meet” OR WebEx OR “Facebook Messenger” OR BlueJeans OR Instagram OR “social media” OR “ICT” OR “information and communications technology”) AND (“senior center*” OR “in place” OR “communit*” OR friend* or famil*) AND (isolat* OR lonel*)

Additionally, analogous MeSH (Medical Subject Headings) terms were applied with PubMed:MEDLINE and EBSCO:MEDLINE databases. MeSH is a controlled vocabulary thesaurus used by the National Library of Medicine (NLM) to index articles, books, and other materials in the biomedical and health sciences.

See Supplementary Table S1 for documentation of the full set of searches.

#### 2.1.5. Process for Study Selection and Data Charting

Records identified from all databases were retrieved and organized in a master Microsoft Excel (Microsoft 365, Microsoft Corp., Redmond, WA, USA) workbook, with a separate worksheet for each source and a consolidated worksheet containing all records. The consolidated worksheet was used to manage study selection. Article attributes saved included article title, author, journal name, publication date, abstract, volume, issue, and page numbers.

Duplicates were first removed, then each of the unique records was reviewed to determine whether one of the exclusion criteria described in Section 2.1.1. (Eligibility Criteria) applied. Once an exclusion criterion was identified, no further evaluation was performed; when multiple exclusion criteria were present, the first criterion noted was assigned. Screening and data charting were conducted by J.A., with independent verification by G.P. or W.S. for the 2021 search and by W.S. for the 2025 update. Discrepancies were resolved through discussion and consensus.

#### 2.1.6. Quality Considerations of Included Studies

The inclusion criteria allowed for primary research that included both qualitative and quantitative studies using either primary or secondary data. To ensure credibility, only peer-reviewed academic journal articles were included. While a formal risk-of-bias assessment is not required for scoping reviews, credibility was supported through this peer-review restriction. Differences in reviewer interpretation during screening or data charting were resolved through discussion and consensus among the research team. This consensus-based approach supports methodological transparency and consistency in the screening and inclusion process, providing an appropriate level of rigor for a scoping review.

#### 2.1.7. Synthesis of Results

The synthesis of data from the studies enabled a logical categorization of findings into themes at both micro- and macro-levels. The review facilitated characterization based on several dimensions, including the type of study, the inclusion of aging-in-place organizations, and the videoconference technologies addressed. To delineate these themes, the researchers employed thematic analysis, a qualitative data analysis technique that supports the classification of topics while ensuring transparency in the coding process. This approach aids in understanding the creation of specific, informative themes. After coding, the codes were evaluated to organize the identified patterns into homogeneously grouped categories.

**Figure 2:**
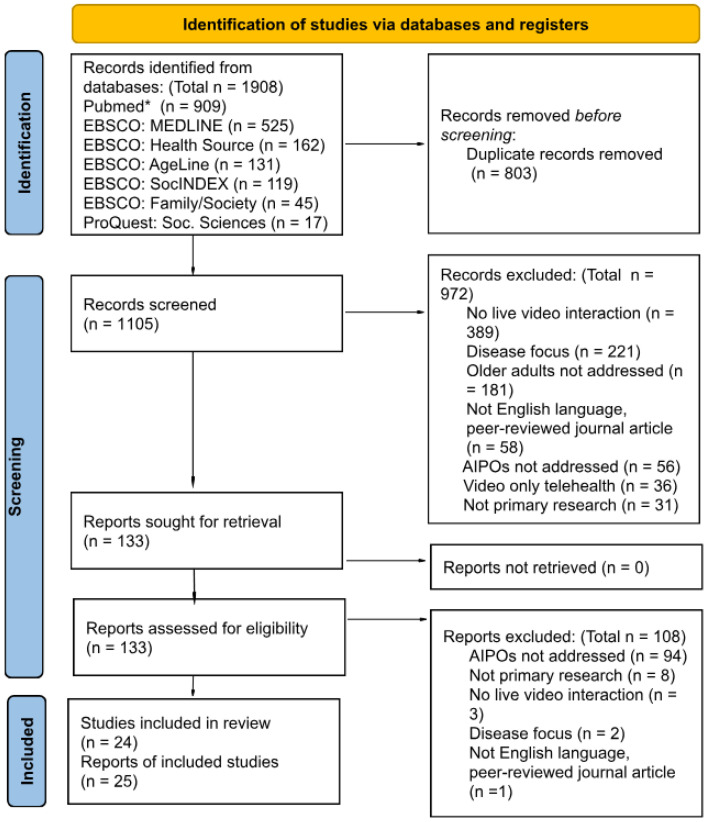
PRISMA 2020 flow diagram of the study selection process. * PubMed (including MEDLINE, in-process, and PubMed Central records).

Data charting and thematic analysis enabled an exploratory mapping of the context, focus areas, and key processes identified within the reviewed literature. A comprehensive and transparent thematic analysis was conducted to organize, explain, and interpret the content, serving as a guide for the structured analysis. Studies were systematically coded into specified categories, classified into themes, and carefully reviewed. Figure 2, presented in the Results section, illustrates the scoping review process, including the stages of identification, screening, eligibility, and inclusion, as well as the categorical dimensions analyzed.

#### 2.1.8. Protocol and Registration

This review was not prospectively registered. A post-registration has been completed on the Open Science Framework (OSF) at https://doi.org/10.17605/OSF.IO/C7AFY.

### 2.2. Latent Dirichlet Allocation (LDA) Analysis

The scoping review was complemented by a thematic analysis using Latent Dirichlet Allocation (LDA) [38], applied to the titles and abstracts of 101 articles identified in the 2021 search. This analysis provided a data-driven overview of the broader literature on relational videoconferencing among older adults, establishing a contextual landscape that situates and informs the subsequent scoping review. LDA is a probabilistic topic modeling technique that elicits topics and topical relationships implicit within a text corpus [42]. RapidMiner software (Version 10.0) [43] was used to perform the LDA analysis.

To apply LDA, portions of records for articles reaching the eligibility stage of the evaluation were copied from the master Microsoft Excel workbook referenced earlier into three separate Microsoft Excel spreadsheet files saved in .csv format. These three files included, respectively, articles published prior to the advent of COVID-19 (35 articles), articles published after the advent of COVID-19 (66 articles), and all articles (101 articles). Together, these represented the 101 articles reaching the eligibility stage. For each article record, a unique article key, publication date, authors, article title, journal name, and abstract were copied into the .csv files.

RapidMiner Studio software (Version 10.0) [43] was applied to each .csv file to perform Latent Dirichlet Allocation based on article title and abstracts. This approach facilitated the identification of potential conceptual groupings within the text corpus, aligning with thematic topics relevant to technology and aging, as guided by the authors’ expertise in these areas. The resulting topic structures were then interpreted in the context of social engagement and aging-in-place organizations, highlighting patterns in technology use that have implications for health, well-being, and intervention design.

### 2.3. Integration of Scoping Review and LDA Findings

The Latent Dirichlet Allocation (LDA) analysis and the scoping review were conducted sequentially and integrated interpretively. LDA was first applied to the 2021 corpus to identify latent topics representing major conceptual domains in the literature on relational videoconferencing among older adults. These data-driven themes informed the analytical framework used in the scoping review, guiding coding and comparison of the thematic content of included studies. Each PRISMA-included study from the full 2011–2025 search was subsequently coded against the LDA-derived themes to assess the extent to which each topic was represented. This integration enabled consideration of thematic trends across different types of aging-in-place organizations (AIPOs), linking implicit thematic patterns in the broader literature with the explicit findings of primary studies.

## 3. Results

Database searches identified 1908 records, with results by database summarized in Table 2. After removing duplicates, 1105 unique records remained (a reduction of 803). Each record was screened to determine whether any exclusion criteria described in Section 2.1.1 (Eligibility Criteria) applied; once an exclusion criterion was met, no further review was conducted. Following the PRISMA reporting flow shown in Figure 2, title and abstract screening excluded 972 records, leaving 133 for full-text assessment. Of these, 108 were excluded based on full-text review, resulting in 25 reports representing 24 studies included in the final scoping review.

The temporal distribution of both records and included studies reflects the evolving landscape of videoconferencing research: of 1105 nonduplicate records screened, 258 (23%) were published between 2011 and 2019, while 847 (77%) were published during 2020–2025, indicating heightened research attention beginning during the COVID-19 pandemic. Included studies, however, were even more uneven, with only 1 of 25 reports (4%) representing a pre-COVID-19 effort.

A total of 972 records were excluded based on title and abstract review. Full texts were retrieved for all remaining records, and 108 were subsequently excluded following full-text evaluation, leaving 25 reports [27,44,45,46,47,48,49,50,51,52,53,54,55,56,57,58,59,60,61,62,63,64,65,66,67] representing 24 unique studies included in the scoping review. Table 3 documents each included study, including key characteristics.

The topic modeling process leveraged the scoping review screening workflow: during the 2021 search, the criteria for primary research and AIPO relevance were applied last. Of the 1105 unique records screened, 558 originated from the 2021 search, forming the broader corpus of 101 peer-reviewed articles addressing social videoconferencing among older adults. This corpus was analyzed using latent topic modeling to define the contextual landscape that informed and guided the subsequent scoping review.

The 101 articles comprising the 2021 topic modeling corpus were further analyzed using structured thematic codes to supplement the automated LDA output. Coding focused on descriptive characteristics such as videoconferencing relationships, organizational involvement, and technology platforms (Table 4). These data provide additional context for interpreting the latent themes and reinforce the relevance of the 2021 corpus as a contextual foundation for the broader scoping review extending through 2025.

### 3.1. Trends

**Figure 3:**
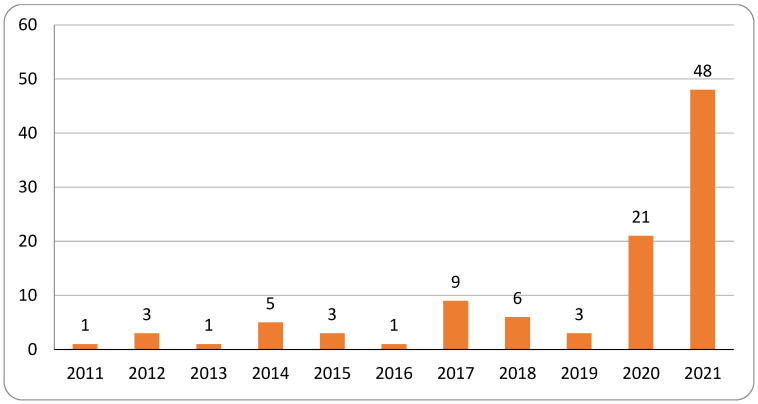
Contextual corpus: report count by year (n = 101).

The 2021 topic modeling and broader contextual analysis revealed a rapid escalation in research on relational videoconferencing among older adults. Publication activity increased sharply beginning in 2020, with that year alone producing nearly as many articles as the entire preceding decade, and 2021 more than doubling the 2020 total (Figure 3). This surge coincides with the onset of the COVID-19 pandemic and the widespread adoption of virtual communication technologies by older populations.

**Figure 4:**
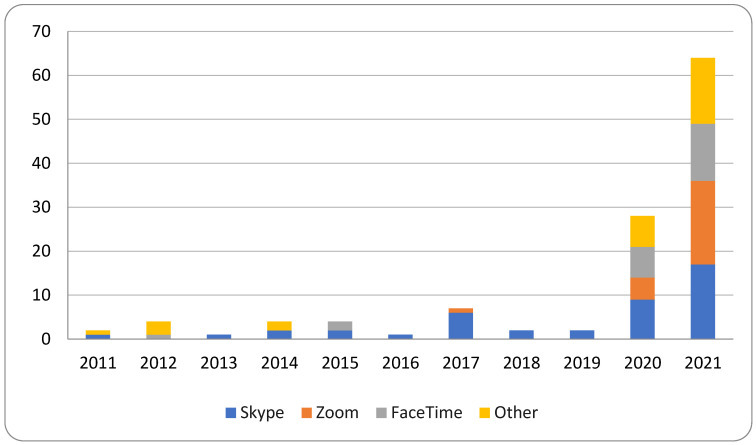
Videoconferencing platforms referenced across the 2021 contextual corpus (n = 101; however, studies may list multiple platforms).

Across the contextual corpus, 67.3% of studies referenced at least one specific videoconferencing platform, with the types cited shifting markedly over time. Skype dominated early references, whereas Zoom and FaceTime saw rapid expansion beginning in 2021, reflecting changes in accessibility, usability, and institutional adoption patterns (Figure 4). Because several studies referenced multiple platforms, cumulative counts exceed the total number of included reports.

**Figure 5:**
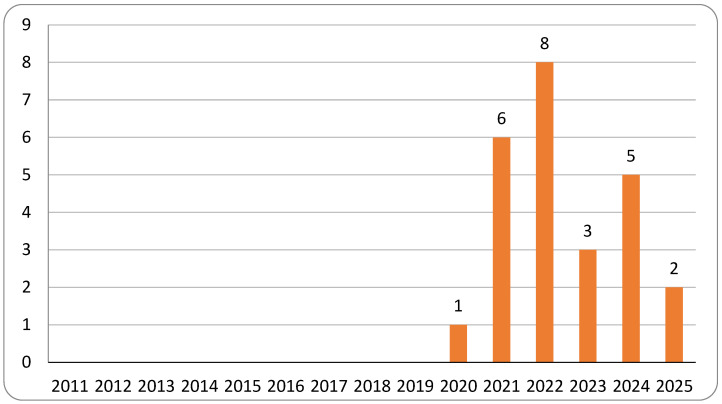
PRISMA-included reports: count by year (n = 25).

**Figure 6:**
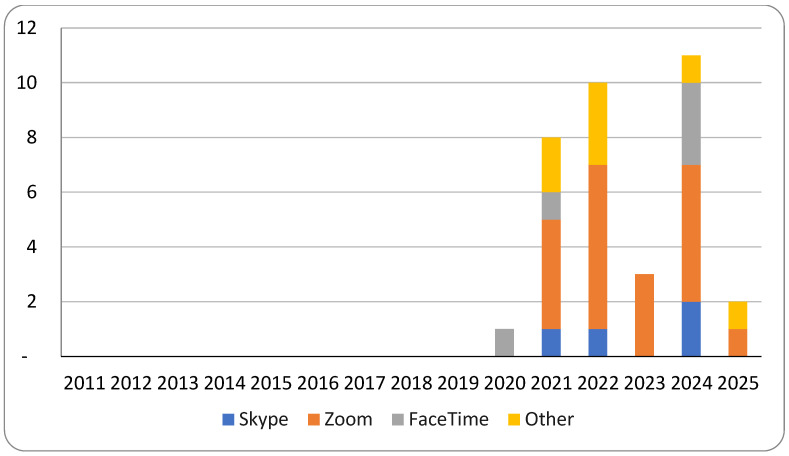
Videoconferencing software referenced across the PRISMA-included studies (n = 24; however, studies may list multiple platforms).

Among the 25 PRISMA-included studies, these temporal patterns were even more pronounced. As illustrated in Figure 5, no eligible studies were published prior to 2020, and publication frequency rose sharply thereafter, mirroring the trend identified in the broader contextual corpus. Following 2021, Zoom became the overwhelmingly dominant platform, while mentions of Skype, FaceTime, and other custom videoconferencing tools declined markedly (Figure 6). This shift suggests increasing convergence toward standardized, accessible technologies in AIPO-facilitated videoconferencing.

### 3.2. Themes

In the contextual corpus, most of the studies investigating relational videoconferencing presented primary data (61.4%), with 7.9% analyzing secondary data (most notably the HRS survey) and 30.7% limited to literature review (see Table 4). The context of the videoconferencing was highly biased toward known individuals (70.3%), with just 9.9% addressing its use to meet new people. Among the articles addressing aging-in-place organizations, the focus was largely on the hosting and connecting roles of those organizations. Mentions of the educational role in supporting adoption were less frequent, and the concept of systematically equipping the nation’s 10,000 senior seniors to support adoption was almost entirely absent. Additionally, Latent Dirichlet analysis (LDA, see Table 5), reveals six emergent topics related to isolation, socialization, physical health, supportive environments, and technology from two different perspectives: technology as intervention and technology as a social medium.

### 3.3. Integration of LDA and Scoping Review Findings

**Figure 7:**
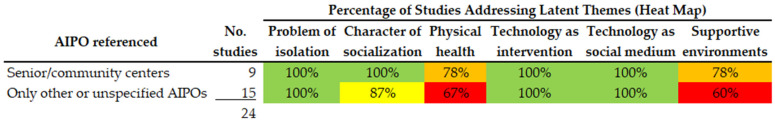
Thematic map of PRISMA-included studies based on latent topics by AIPO type. Colors indicate the percentage of studies addressing each theme: green (90–100%), yellow (80–<90%), orange (70–<80%), and red (<70%).

The integration of LDA and scoping review findings allowed for an examination of how the six LDA-derived themes were reflected across different AIPO types. While all thematic domains were represented to some degree, Physical Health and Supportive Environments were addressed less consistently than other themes, particularly among studies that did not reference senior or community centers. This pattern suggests that AIPO-led videoconferencing initiatives often emphasize social connection and engagement more than environmental or physical health dimensions. The distribution of themes by AIPO type is visualized in Figure 7 (Thematic map of PRISMA-included studies based on latent topics by AIPO type), and detailed mappings for each study are presented in Supplementary Table S2 (Mapping of latent topics to PRISMA-included studies).

## 4. Discussion

### 4.1. Main Conclusions

The use of videoconference technology to enhance the social engagement efforts of aging-in-place organizations was found to be understudied in the literature, despite their mandate and wide geographic penetration in the U.S., even considering literature published well into the COVID-19 pandemic. As noted earlier, older adults’ digital engagement has been found to depend on structured support, further underscoring AIPOs’ role as facilitators of adoption [15]. Recent prospective evidence likewise shows that low-tech, structured telemedicine programs can improve outcomes for older adults [68]. Non-academic sources address the topic more specifically, reflecting a developing need and practice for which there is a clear gap in the literature. We found the phrase “relational videoconference technology” (or any variations beginning with “relational video”) entirely absent from the abstracts identified in the literature search, and we suggest that our use here introduces the term as a tool to distinguish relational videoconference use and design from telemedical use and design [69] as well as from text and audio forms of synchronous communication [70].

We paired a scoping review with unsupervised Latent Dirichlet Allocation (LDA) topic modeling: the review provides a transparent, question-driven synthesis, while LDA maps latent themes and their prevalence across the broader literature. The scoping review findings and LDA analysis reflect complementary yet distinct insights into the role of videoconferencing for older adults’ social engagement. Both approaches underscore growing interest in videoconferencing technologies, particularly during the COVID-19 pandemic, and highlight the potential of such tools in mitigating social isolation among older adults.

The expanded 2025 search substantially broadened the evidence base, identifying a new wave of post-pandemic studies that document enduring, community-based uses of videoconferencing by aging-in-place organizations. These newer studies describe sustained or hybrid models—such as intergenerational mentoring, creative arts, and storytelling programs—implemented through community centers, libraries, and Area Agencies on Aging. Together, they demonstrate a gradual shift from temporary pandemic adaptations toward more institutionalized strategies for digital social engagement among community-dwelling older adults.

### 4.2. Interpretation in Relation to Previous Work

The scoping review reveals patterns and directions in the literature, providing a structured summary and analysis of the research on relational videoconferencing. It highlights the important role of aging-in-place organizations in aiding adoption but identifies a notable gap regarding methodical strategies for facilitating widespread adoption. Additionally, the review uncovers a bias in the relevant studies toward facilitating existing relationships, such as friends and family, as opposed to forming new relationships through videoconferencing in the wake of COVID-19, but expanding to include new relationships in more recent studies. A key success of this study is its illumination of the practical challenges and opportunities in leveraging videoconferencing within this demographic.

Compared with earlier findings, the updated corpus suggests that AIPO engagement is no longer anecdotal but emerging as a small yet identifiable domain of practice. The literature now offers concrete examples of structured community programs, though systematic evaluation and replication remain limited. This evolution reframes the landscape from one of sparse documentation to one of formative evidence, where the challenge lies less in invention than in codification and comparison of existing models with a focus on opportunities to replicate and scale.

### 4.3. Complementarity of Methods: Integrating Scoping Review with Computational Text Analysis

The LDA analysis contrasts with the scoping review by exploring thematic relationships in the literature and identifying emergent topics. Examples of these topics include isolation, socialization, the dual perspectives of technology as a social medium and as an intervention, and physical health. Distinct from the scoping review, LDA provides probabilistic insights into how these topics interconnect, revealing implicit relationships and aspects not readily apparent through a scoping literature review. For example, LDA reveals dual roles for technology and a framework that encompasses applications in both direct interventions and the promotion of supportive environments.

While the scoping review emphasizes cataloging explicit findings, LDA goes deeper by revealing latent themes and relationships. However, there is an important difference in their emphasis: the scoping review details practical applications as well as gaps in the literature, while LDA lends insight into the role of videoconferencing in the social engagement of older adults. Together, these approaches provide complementary bases confirming the potential videoconferencing holds for addressing loneliness and social isolation, even as they differ in focus, with one utilizing explicit evidence and the other revealing implicit themes.

### 4.4. Implications for Practice and Policy

Near-term practice should focus on documenting what is already happening. Rather than prescribing detailed programs, AIPOs and their partners can begin by systematically describing current practices—how devices, data, training, spaces, and facilitation are being arranged locally—and by tracking a small set of streamlined measures (e.g., reach, first-use success, early retention, user-reported barriers). Comparing these practices across organizations can reveal patterns worth testing while still allowing for local variability.

### 4.5. Research Priorities, Strengths, and Limitations

The research priority is to build the evidence base before scaling solutions. Early studies should emphasize environmental scans, descriptive surveys, multiple-case (realist) studies, and process evaluations that clarify who is doing what, for whom, in which contexts, and with what obstacles and workarounds. Mixed-methods designs that pair basic usage/engagement indicators with qualitative accounts of confidence, trust, and support will help identify existing approaches for further evaluation. Where theory is applied, adoption models (e.g., UTAUT-2; COM-B) and implementation/normalization frameworks (e.g., CFIR; NPT) can serve as organizing lenses, using a minimal set of constructs to guide measurement and reporting without prescribing program content [23,71,72,73].

To minimize diffusion of responsibility while maintaining flexibility, AIPOs lead local documentation and basic monitoring; researchers partner to design comparable measures and explain variation; policymakers/funders enable flexible, small-scale pilots with simple reporting; and platform designers support partners with accessible technology and privacy-respecting usage summaries. These roles keep the focus on learning what works rather than assuming it.

Researchers should ensure terminology is clear: when using the term “recommendation,” they should denote near-term priorities for documenting and evaluating practice, not fixed, one-size-fits-all programs. Taken together, this staged approach—describe → compare → refine → test—positions the field to move from promise to practice. Accumulating evidence will allow for more specific interventions and policies. For now, the most valuable step is to make visible the work already being done, learn from it, and build the shared concepts and measures that will sustain cumulative progress.

Strengths of this study include its hybrid methodology combining a scoping review with computational text analysis, its specific focus on the understudied context of aging-in-place organizations (AIPOs), transparent reporting following PRISMA-ScR guidelines, and a systematic approach to study selection and data extraction. This combination enables both contextual breadth and methodological rigor while identifying critical evidence gaps.

This study synthesizes a limited body of literature on the role of AIPOs in relational videoconferencing. Using a hybrid methodology (scoping review plus topic modeling), it identifies patterns, thematic structures, and latent relationships within the literature but does not evaluate intervention effectiveness or support generalization beyond the studies reviewed.

The review was limited to studies published in English, which may have excluded relevant research from non-English-language sources. As a result, the findings may reflect an Anglophone bias and may limit generalizability regarding cultural or regional variations in aging-in-place practices or technology adoption. Additionally, the exclusion of grey literature may have omitted relevant insights from unpublished programs, implementation reports, and community-based innovations. While the included peer-reviewed studies provide a sufficient evidence base for mapping the landscape, this exclusion may limit the comprehensiveness of the review.

### 4.6. Future Research Directions

Future research should prioritize longitudinal tracking, cross-organizational comparisons, and systematic evaluation of implementation strategies. Developing a shared framework for documentation, concepts, and measures will accelerate cumulative knowledge and enable progression from descriptive mapping to rigorous intervention assessment.

## 5. Conclusions

Relational videoconferencing holds promise for sustaining, and in some contexts expanding, older adults’ social connections. AIPOs have a broad last-mile reach and a public mandate to support aging in place, positioning them as natural hosts and coordinators for relational videoconferencing initiatives. Yet our review shows that the role of AIPOs in enabling adoption remains sparsely documented and under-theorized. By integrating a scoping review with unsupervised topic modeling, we mapped both what is known and how the literature clusters, and the picture that emerges is less a mature intervention space than a formative learning environment in which practices are evolving more rapidly than the scholarship describing them.

AIPOs should focus on documenting and supporting older adults’ use of relational videoconferencing by providing structured guidance, technical facilitation, and accessible training. Simple, consistent tracking of engagement and early outcomes can reveal patterns and inform iterative improvements without prescribing rigid programs.

Future studies should prioritize descriptive and longitudinal designs, cross-organizational comparisons, and the development of shared frameworks for measurement and documentation. Emphasis on understanding who uses these technologies, in what contexts, and with what barriers will strengthen the evidence base needed for effective interventions. The field’s priority is shifting from discovering whether community-led videoconferencing can enhance social connection to understanding how, for whom, and under what organizational conditions these practices can be sustained and scaled.

## Figures and Tables

**Table 1:** Key terms.

Term	Working Definition	Key Sources
Social isolation	An objective state of being alone characterized by limited social ties and infrequent or low-quality contact with others.	Hansen et al. (2024) [30]; see also de Jong-Gervield (1985) [31]
Loneliness	A subjective state of feeling alone characterized by a perceived gap between desired and actual social connections.	Hansen et al. (2024) [30]; see also de Jong-Gervield (1985) [31]
Social technology	Digital tools (both software and hardware) that enable interpersonal interaction and shape social processes.	Byrne et al. (2021) [32]; Chopik (2016) [33]; Ten Bruggencate et al. (2019) [34]
Aging-in-place organizations (AIPOs)	Community-based entities that support older adults to live safely and independently at home by providing/coordinating long-term services and supports (LTSS), information/navigation, and social connection.	Older Americans Act (1965) [25]

**Table 2:** Results by database.

Database	Records
PubMed:MEDLINE	909
EBSCO:MEDLINE	525
EBSCO: Health Source:Nursing/Academic Edition	162
EBSCO:SocINDEX	119
EBSCO:AgeLine	131
EBSCO:Family & Society Studies Worldwide	45
ProQuest:Social Science Premium Collection	17
All databases	1908

**Table 3:** Characteristics of included studies.

Report No.	Author (Year)	Country	Relationships Context	Pre/Post COVID-19	Videoconference Technology	Type of AIPO
1	(Fields et al., 2020) [52]	USA	Nonspecified	Pre	FaceTime	Community-based organization (home visits)
2	(Cohen-Mansfield et al., 2021) [47]	Israel	Existing relationships	Post	Zoom	Senior centers
3	(Greenwood-Hickman et al., 2021) [63]	USA	Existing relationships	Post	FaceTime, Zoom	Community-based organizations (unspecified)
4	(Jiménez et al., 2021) [46] *	USA	New relationships	Post	Well Connected	Community-based organizations (including home meal delivery)
5	(Marmo et al., 2021) [27]	USA	Existing relationships	Post	Nonspecified	Senior center
6	(Sanchez-Villagomez et al., 2021) [60]	USA	Existing relationships	Post	Skype, Zoom	Community-based organizations (unspecified)
7	(Shapira et al., 2021) [44]	Israel	New relationships	Post	Zoom	Community-based organizations (“social care”)
8	(Appel et al., 2022) [67]	Canada	New and existing relationships	Post	Jitsi	Library
9	(Beauchet et al., 2022) [48]	Canada	New relationships	Post	Zoom	Art museum
10	(Gadbois et al., 2022) [51] *	Additional report on the same study as Report 4 [46]				
11	(Gray et al., 2022) [58]	Canada	New relationships	Post	Zoom	Community-based organizations (unspecified)
12	(Juris et al., 2022) [65]	USA	New and existing relationships	Post	Zoom	Area Agency on Aging, senior centers, community-based organizations (including home meal delivery)
13	(O’Connell et al., 2022) [56]	Canada	New and existing relationships	Post	Google Meet, Zoom	Community-based organizations (unspecified)
14	(Roberts et al., 2022) [53]	USA	New relationships	Post	Zoom	Senior centers, other community-based organizations (unspecified)
15	(Strutt et al., 2022) [61]	Australia	Existing relationships	Post	Microsoft Teams, Skype, Zoom	Community-based organizations (unspecified)
16	(Vega et al., 2023) [49]	USA	New and existing relationships	Post	Zoom	Area Agency on Aging, senior center, other community-based organizations
17	(Weselman et al., 2023) [54]	Australia	New relationships	Post	Zoom	Community-based organizations (unspecified)
18	(Wolman et al., 2023) [55]	Canada	Existing relationships	Post	Zoom	Community center
19	(Grey et al., 2024) [62]	United Kingdom	Existing relationships	Post	FaceTime, Skype, Zoom	Community-based organizations (adult social care)
20	(Nguyen-Truong et al., 2024) [64]	USA	Existing relationships	Post	Facebook Messenger, FaceTime, FCC HD, Google Duo/Google Meet, KakaoTalk, LINE, Skype, Tango, Telegram, Viber, WeChat, WhatsApp, Zalo, Zoom	Senior center
21	(Steinman et al., 2024) [59]	USA	New relationships	Post	Zoom	Aging service organization
22	(Sun et al., 2024) [50]	Canada	Existing relationships	Post	Zoom	Senior centers
23	(Tsotsoros et al., 2024) [57]	USA	New and existing relationships	Post	FaceTime, Zoom	Senior/community centers
24	(Mois et al., 2025) [45]	USA	New relationships	Post	OneClick	Community-based organizations (unspecified)
25	(Pollak et al., 2025) [66]	USA	New relationships	Post	Zoom	Community-based organization (unspecified)

* Reports 4 and 10 are distinct publications on the same study.

**Table 4:** Characteristics of the 2021 contextual corpus (% of 101 articles on relational videoconferencing by older adults).

Aspect	Rate of Occurrence
Videoconferencing with known persons	70.3%
Videoconferencing with unknown persons	9.9%
Videoconferencing with unspecified persons	13.9%
Videoconferencing supported by local organizations	22.8%
Type of article: literature search	30.7%
Type of article: primary data	61.4%
Type of article: secondary data	7.9%
Mention specific videoconference platform	67.3%
Mention Skype	42.6%
Mention Zoom	24.8%
Mention FaceTime	22.8%
Mention Google (Meet/Duo/Hangouts)	5.9%
Mention custom videoconference application	5.0%
Mention WhatsApp video calling	3.0%
Mention Facebook Messenger	3.0%
Mention other videoconference platforms	10.9%

**Table 5:** LDA: emergent topics and key terms.

Problem of Isolation	Character of Socialization	Physical Health	Technology as Intervention	Technology as Social Medium	Supportive Environments
Interventions	Group	COVID	ICT	Social	Residents
Studies	Activities	Pandemic	Loneliness	Older	Care
Review	Interest	Older	May	Technology	Nursing
Isolation	Training	Adults	Use	Adults	Homes
People	Interactions	Health	Users	Use	Loneliness

## Data Availability

The raw data supporting the conclusions of this article will be made available by the authors upon request.

## Supplementary Materials

The following supporting information can be downloaded at: https://www.mdpi.com/article/10.3390/healthcare13233031/s1, Table S1: Scoping review search strategy; Table S2: Mapping of latent topics to PRISMA-included studies.
