# Supplementary material for: Hybrid Analysis of Videoconference Technology Use by Aging-in-Place Organizations to Promote Social Engagement for Older Adults: A Scoping Review with Latent Topic Modeling
Source: Healthcare (Basel). 2025 Nov 24;13(23):3031. doi: 10.3390/healthcare13233031 (PMC12692519; doi:10.3390/healthcare13233031)
Supplement: Supplementary file 1 [file healthcare-13-03031-s001.zip › 02. Supplementary Table S2 Mapping of Latent Topics to PRISMA-Included Studies v2.docx]

**Supplementary Table S2:** Mapping of Latent Topics to PRISMA-Included Studies

| **Report No.** | **Author (Year)** | **Problem of**  **Isolation** | **Character of**  **Socialization** | **Physical health** | **Technology as Intervention** | **Technology as Social Medium** | **Supportive Environments** |
| --- | --- | --- | --- | --- | --- | --- | --- |
| 1 | (Fields et al., 2020) [52] | **✓** |  | **✓** | **✓** | **✓** |  |
| 2 | (Cohen-Mansfield et al., 2021) [47] | **✓** | **✓** | **✓** | **✓** | **✓** | **✓** |
| 3 | (Greenwood-Hickman et al., 2021) [63] | **✓** | **✓** |  | **✓** | **✓** |  |
| 4 | (Jiménez et al., 2021) [46]* | **✓** | **✓** | **✓** | **✓** | **✓** | **✓** |
| 5 | (Marmo et al., 2021) [27] | **✓** | **✓** | **✓** | **✓** | **✓** |  |
| 6 | (Sanchez-Villagomez et al., 2021) [60] | **✓** | **✓** |  | **✓** | **✓** | **✓** |
| 7 | (Shapira et al., 2021) [44] | **✓** | **✓** | **✓** | **✓** | **✓** |  |
| 8 | (Appel et al., 2022) [67] | **✓** | **✓** | **✓** | **✓** | **✓** | **✓** |
| 9 | (Beauchet et al., 2022) [48] | **✓** | **✓** | **✓** | **✓** | **✓** |  |
| 10 | (Gadbois et al., 2022) [51]* | **Additional report on same study as Report 4 (46)** | | | | |  |
| 11 | (Gray et al., 2022) [58] | **✓** | **✓** | **✓** | **✓** | **✓** | **✓** |
| 12 | (Juris et al., 2022) [65] | **✓** | **✓** |  | **✓** | **✓** | **✓** |
| 13 | (O’Connell et al., 2022) [56] | **✓** | **✓** | **✓** | **✓** | **✓** |  |
| 14 | (Roberts et al., 2022) [53] | **✓** | **✓** | **✓** | **✓** | **✓** |  |
| 15 | (Strutt et al., 2022) [61] | **✓** |  | **✓** | **✓** | **✓** |  |
| 16 | (Vega et al., 2023) [49] | **✓** | **✓** | **✓** | **✓** | **✓** | **✓** |
| 17 | (Weselman et al., 2023) [54] | **✓** | **✓** | **✓** | **✓** | **✓** | **✓** |
| 18 | (Wolman et al., 2023) [55] | **✓** | **✓** | **✓** | **✓** | **✓** | **✓** |
| 19 | (Grey et al., 2024) [62] | **✓** | **✓** |  | **✓** | **✓** | **✓** |
| 20 | (Nguyen-Truong et al., 2024) [64] | **✓** | **✓** | **✓** | **✓** | **✓** | **✓** |
| 21 | (Steinman et al., 2024) [59] | **✓** | **✓** | **✓** | **✓** | **✓** | **✓** |
| 22 | (Sun et al., 2024) [50] | **✓** | **✓** | **✓** | **✓** | **✓** | **✓** |
| 23 | (Tsotsoros et al., 2024) [57] | **✓** | **✓** |  | **✓** | **✓** | **✓** |
| 24 | (Mois et al., 2025) [45] | **✓** | **✓** |  | **✓** | **✓** | **✓** |
| 25 | (Pollak et al., 2025) [66] | **✓** | **✓** |  | **✓** | **✓** | **✓** |
| ^*^ Reports 4 and 10 are distinct publications on the same study | | | | | | | |
